# CD36 gene variants and their potential in determining T2DM susceptibility

**DOI:** 10.1186/1755-8166-7-S1-P39

**Published:** 2014-01-21

**Authors:** Monisha Banerjee, Sunaina Gautam, CG Agrawal

**Affiliations:** 1 Molecular & Human Genetics Laboratory, Department of Zoology, University of Lucknow, India; 2 Department of Medicine, King George’s Medical University, Lucknow, India

## Background

Type 2 diabetes (T2DM) is a non-communicable disease affecting huge populations in India. Several groups all over the world are in search of prognostic genetic markers for the early detection of T2DM. Single nucleotide polymorphisms (SNPs) in CD36, a macrophage scavenger receptor were implicated in the pathogenesis of T2DM and its complications. This molecule is responsible for the uptake of free fatty acids specially oxidized low density lipoprotein (Ox-LDL) during diseased conditions.

Aim of the present study was to find out the allelic and genotypic frequencies of 11 SNPs spanning the entire CD36 gene in north Indian population and find their association with T2DM. In addition, haplotypic analysis was undertaken to find out the risk haplotype in individuals of families with a history of T2DM.

## Material and methods

All the 11 SNPs were genotyped in at least 100 each of controls and T2DM subjects using polymerase chain reaction-restriction fragment length polymorphism (PCR-RFLP) and single-strand conformation polymorphism (SSCP) methods. Haplotype analysis was done by SHEsis software. Ten families with diabetic history were identified and blood samples were collected from available family members. DNA was extracted by salting out method and three SNPs viz. rs1761667 (G>A) in exon 1A, rs3211938 (T>G) in exon 10 and rs3212018 (16 bp del) in exon 14 were genotyped in all individuals. Anthropometric characteristics viz. systolic/diastolic blood pressures, body mass index (BMI), waist hip ratio (WHR) and biochemical parameters were measured in all individuals.

## Results

Out of 11 SNPs, six were polymorphic (rs1984112, A>G; rs1761667, G>A; rs1527479, C>T; rs3211938, T>G; rs1527483, C>T; rs3212018, 16bp del) in the study population. Individuals having a haplotypic combination ‘AACGC1’ showed highest significant association with T2DM (P<0.001). Family studies showed that individuals with risk genotype ‘GA’ of rs1761667, ‘G’ allele of rs3211938 (T>G) or ‘G’ allele of rs1984112 (T>G) had an increased BMI and relatively higher glucose levels.

## Conclusions

Therefore, we conclude that haplotypes/genotypes/alleles of the CD36 gene variants can be potential markers in determining diabetes risk and such analyses may be useful for early identification of individuals susceptible to T2DM and its complications in the north Indian population.

